# Supplementary material for: Multifunctional non-woven fabrics of interfused graphene fibres
Source: Nat Commun. 2016 Nov 30;7:13684. doi: 10.1038/ncomms13684 (PMC5141476; doi:10.1038/ncomms13684)
Supplement: Supplementary Information — Supplementary Figures 1-16, Supplementary Tables 1-5, Supplementary Note 1 and Supplementary References [file ncomms13684-s1.pdf]

a

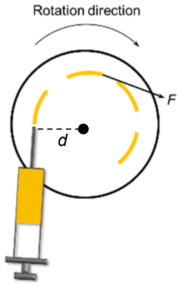

b

Code	1	2	3	4
Injecting speed (mm min^{-1})	0.3	0.2	0.2	0.1
Rotating speed (r min^{-1})	42	42	42	42
Nozzle position (*d*) (mm)	40	40	20	40

c **d**

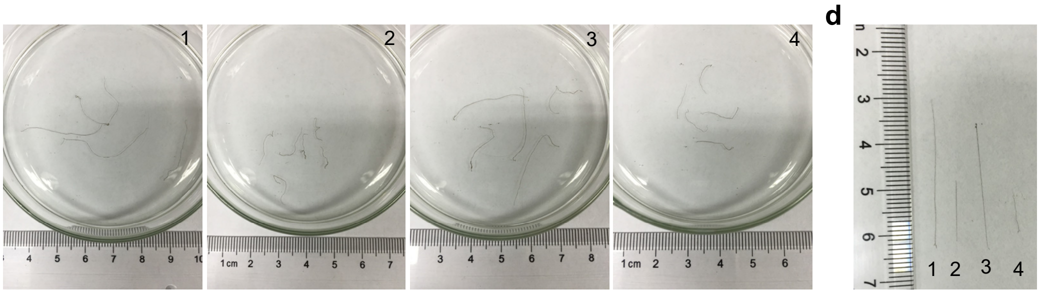

Supplementary Figure 1 (**a**) Schematic illustration showing continuous fabrication of GO staple fibres by *in-situ* stretching. The coagulation bath rotated rapidly to elongate the as-extruded gel fibres in the rotation direction until they break. The friction force *F* between gel fibres and the liquid is the driving force for the so-called *in situ* stretching. (**b**) Parameters for 4 types of GO fibres with different lengths. Photographs of (**c**) the corresponding as-spun GO fibres and (**d**) the dried GO fibres.

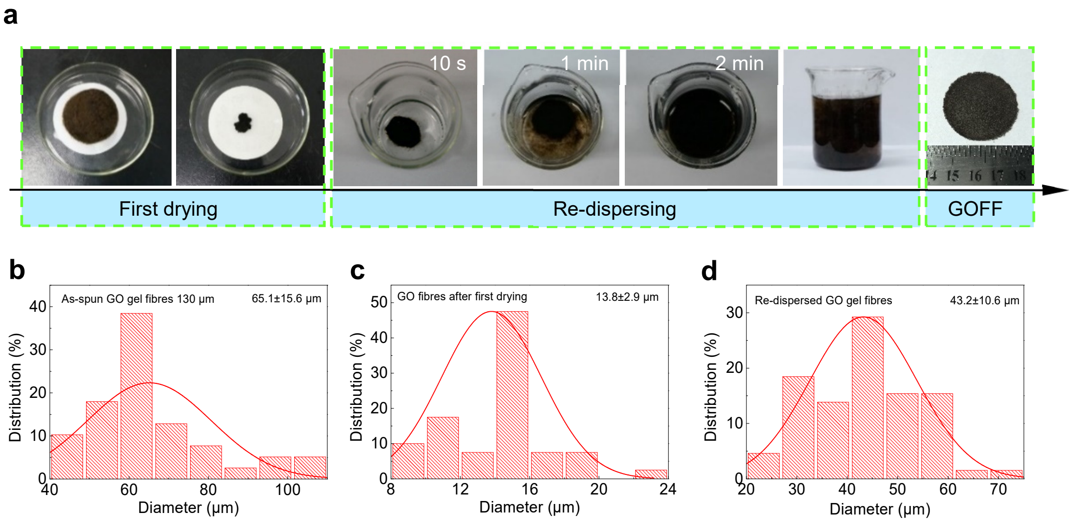

Supplementary Figure 2 (**a**) Photographs recording the first drying and re-dispersing processes and the resultant GOFF after re-dispersing and drying. The variation of fibre diameter for (**b**) as-spun GO gel fibres, (**c**) solid GO fibres after first drying and (**d**) re-dispersed GO gel fibres. A 130 μm spinneret was used. The smaller fibre diameter of re-dispersed fibres indicates reduced solvent content within the fibres compared with the as-spun ones, which is called the confined swelling. Therefore the shrinkage during the second drying was significantly reduced.

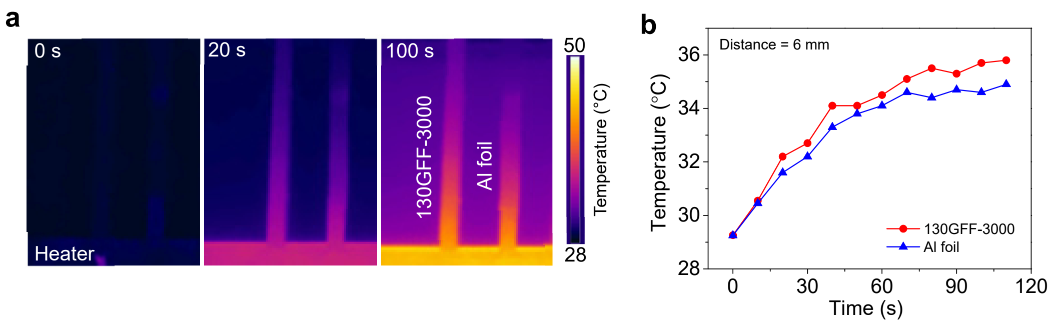

Supplementary Figure 3 (**a**) Thermal transport evolution of 130GFF-3000 and Al foil. Samples with the same size and thickness were spray-coated with graphite paint to get the same emissivity under the infrared camera. (**b**) Temperature on the samples as a function of heating time. Temperature was measured at the midpoint of each sample (6 mm away from the heater). This demonstrates that the in-plane thermal conductivity of 130GFF-3000 is higher than that of Al foil (237 W m^{-1} K^{-1}).

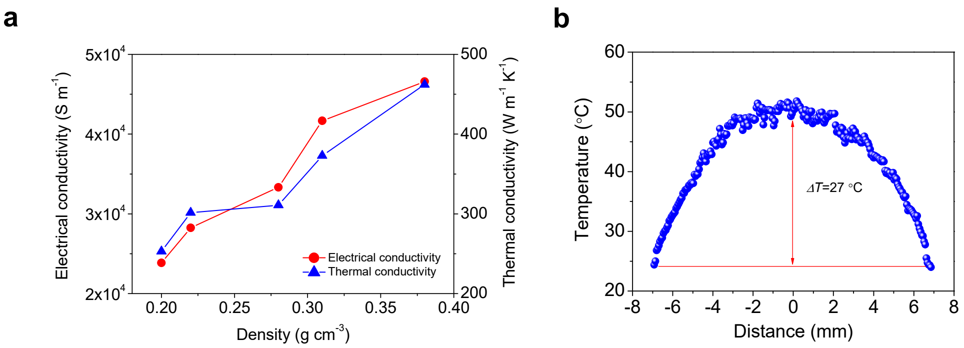

Supplementary Figure 4 (**a**) Electrical and thermal conductivities of 130GFFs as a function of density. (**b**) Temperature profile along a thin strip of 130GFF-3000 for thermal conductivity measurement using the self-heating method. According to the equation $\kappa = UIL/4A\Delta T$, where κ, U, I, L, A and ΔT are thermal conductivity, voltage, current, half length of the strip sample, cross-section area of the sample and temperature difference between the midpoint and the ends of the sample, respectively, the thermal conductivity of the 130GFF-3000 sample is calculated to be 301.5 W m^{-1} K^{-1}.

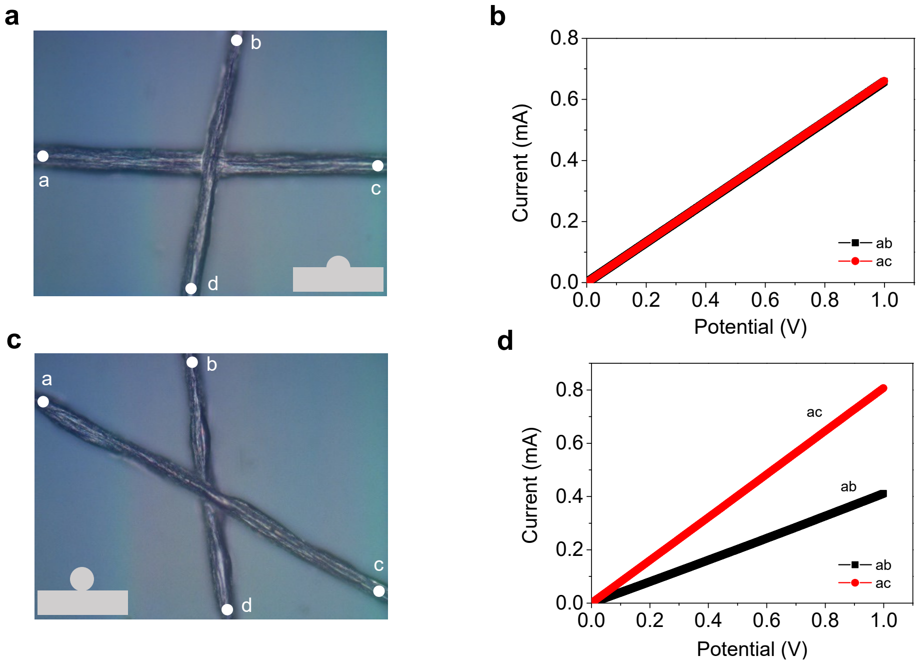

Supplementary Figure 5 Micrographs of (**a**) crossed graphene fibres with fused junction and (**c**) simply overlapped graphene fibres with unfused junction. Insets illustrate the side view of the junctions. I-V curves of ab and ac in (**b**) fused graphene fibres and (**d**) unfused graphene fibres. The resistance at junction is negligible in the fused ones.

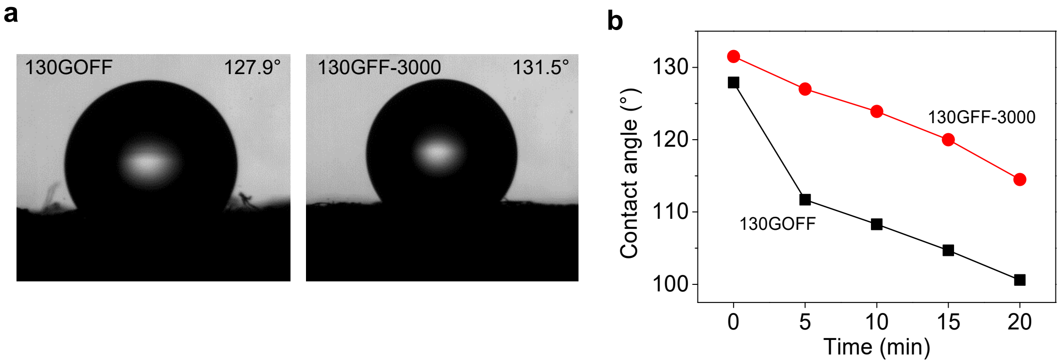

Supplementary Figure 6 (**a**) Water contact angle of 130GOFF (left) and 130GFF-3000 (right). (**b**) Water contact angle of 130GOFF and 130GFF-3000 with respect to time. While keeping a water droplet (2 μL) on top of the fabrics, the contact angle gradually decreased over time. Both the original GOFF and annealed GFF followed the same trend owing to water penetration caused by gravity, and additional water-absorption from hydrophilic GO fibres in GOFF.

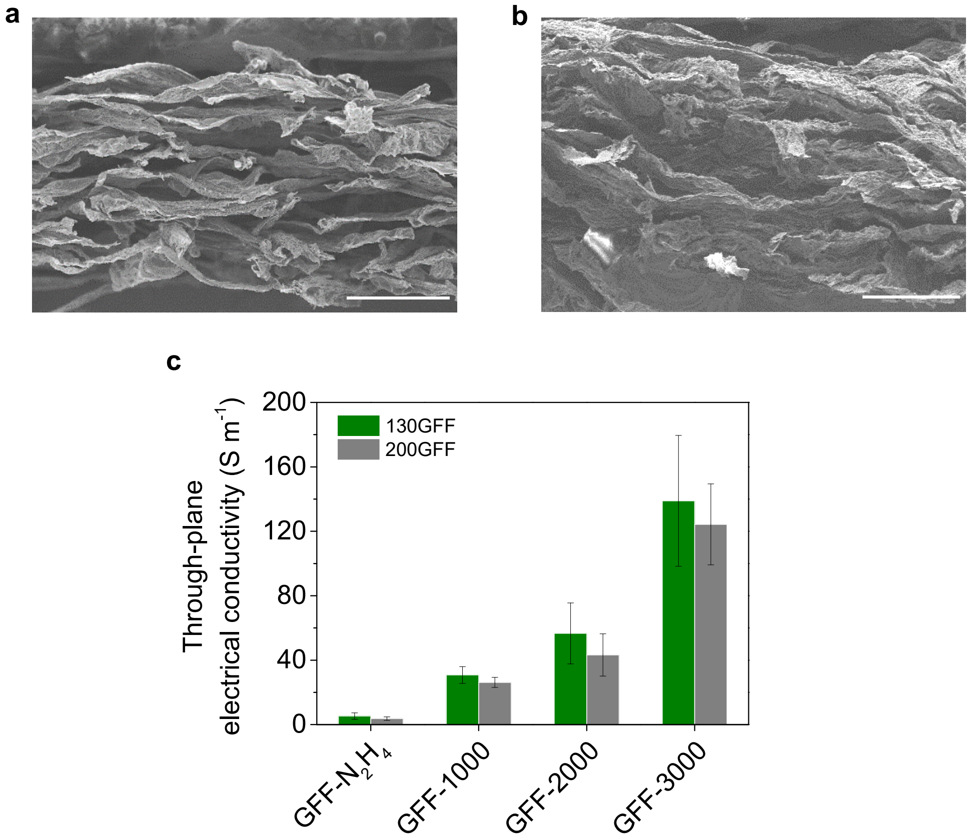

Supplementary Figure 7 SEM images of the cross-section of (**a**) 130GFF-3000 and (**b**) 200GFF-3000. (**c**) Through-plane electrical conductivities of 130GFFs and 200GFFs. Scale bars, 100 µm. The anisotropic conductivity of GFFs could be attributed to two reasons. First, the conductivity of graphene fibres is anisotropic because of the well-aligned graphene sheets along the axial direction. Second, the graphene fibres are mostly oriented in the in-plane direction of GFFs and loosely packed through the thickness, thus form an anisotropic network structure. Error bars represent the standard deviation of the through-plane electrical conductivity of different GFF samples.

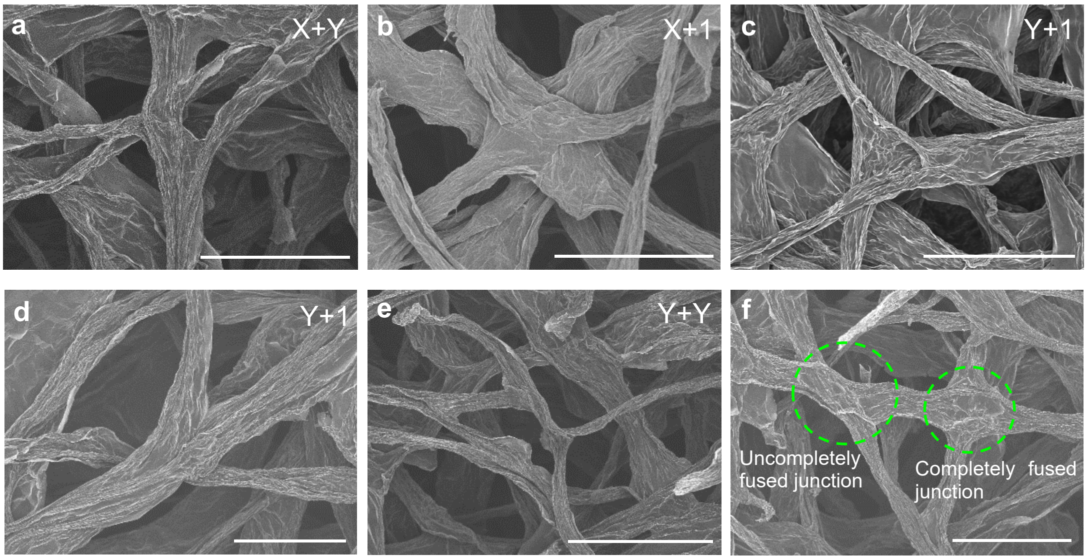

Supplementary Figure 8 SEM images showing examples for complex junctions: (**a**) (X+Y)-type junction, (**b**) (X+1)-type junction (an X-type junction connected with a graphene fibre), (**c**) (Y+1)-type junction, (**d**) another kind of (Y+1)-type junction and (**e**) (Y+Y)-type junction. (**f**) SEM images indicating the difference between uncompletely and completely fused junctions. The boundary between two fibres is much clearer for the uncompletely fused junction. Scale bars, 100 µm (**a-c**, **e**, **f**) and 50 µm (**d**).

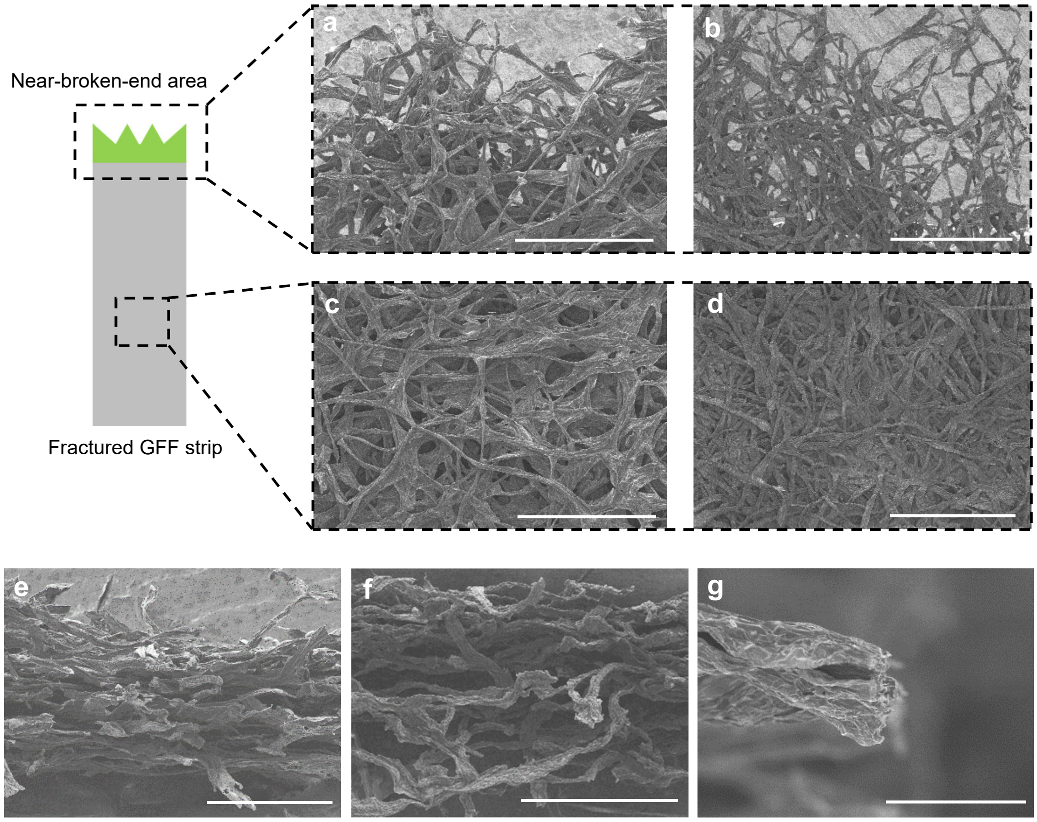

Supplementary Figure 9 The loose morphology of near-broken-end areas of (**a**) 130GFF-3000 and (**b**) 200GFF-3000 after tensile tests. It is much looser in 200GFF-3000 near the broken end, due to a weaker inter-fibre connection. The relatively dense areas away from the broken ends in (**c**) 130GFF-3000 and (**d**) 200GFF-3000. Cross-sectional morphology of the broken ends in (**e**) 130GFF-3000 and (**f**) 200-GFF-3000. (**g**) A graphene fibre pulled out from the broken end of 200GFF-3000. Scale bars, 400 μm (**a, c**), 1 mm (**b, d**), 200 μm (**e**), 500 μm (**f**) and 40 μm (**g**)

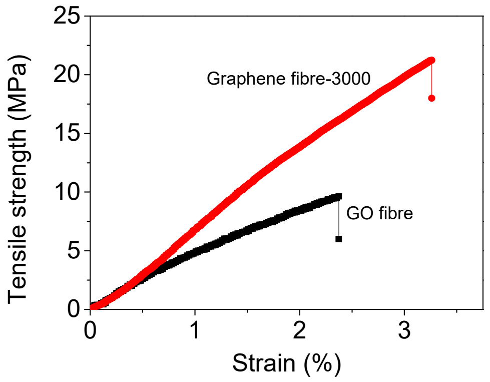

Supplementary Figure 10 Stress-strain curves of individual GO staple fibre and graphene staple fibre after 3000 °C annealing.

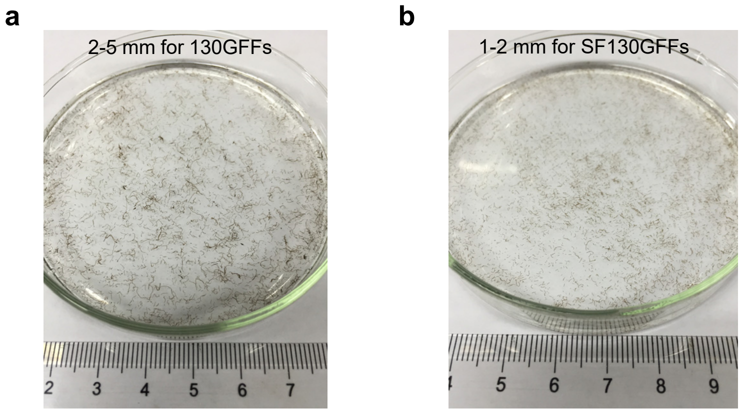

Supplementary Figure 11 Photographs of re-dispersed GO staple fibres with different length for preparation of (**a**) 130GFFs and (**b**) SF130GFFs (130GFFs made from shorter fibres).

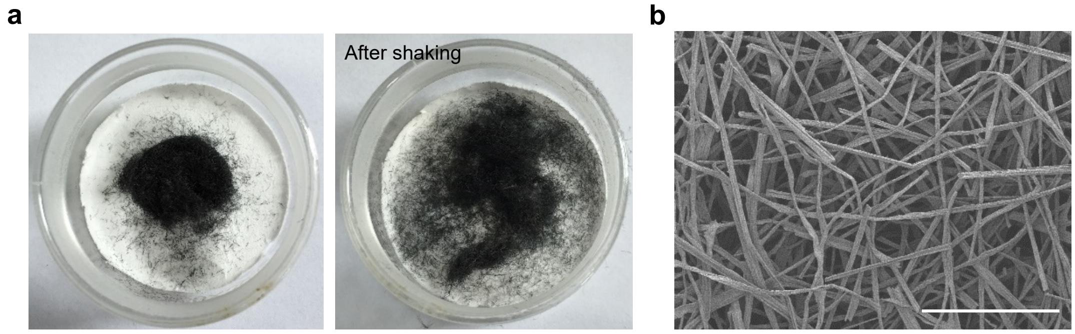

Supplementary Figure 12 (**a**) Photographs of unfused graphene fibres (3000 °C annealed) before (left) and after (right) mild shaking, indicating that the short graphene fibres cannot form stable structures which would fall apart easily. (**b**) SEM image of unfused graphene staple fibres. Scale bar, 500 μm.

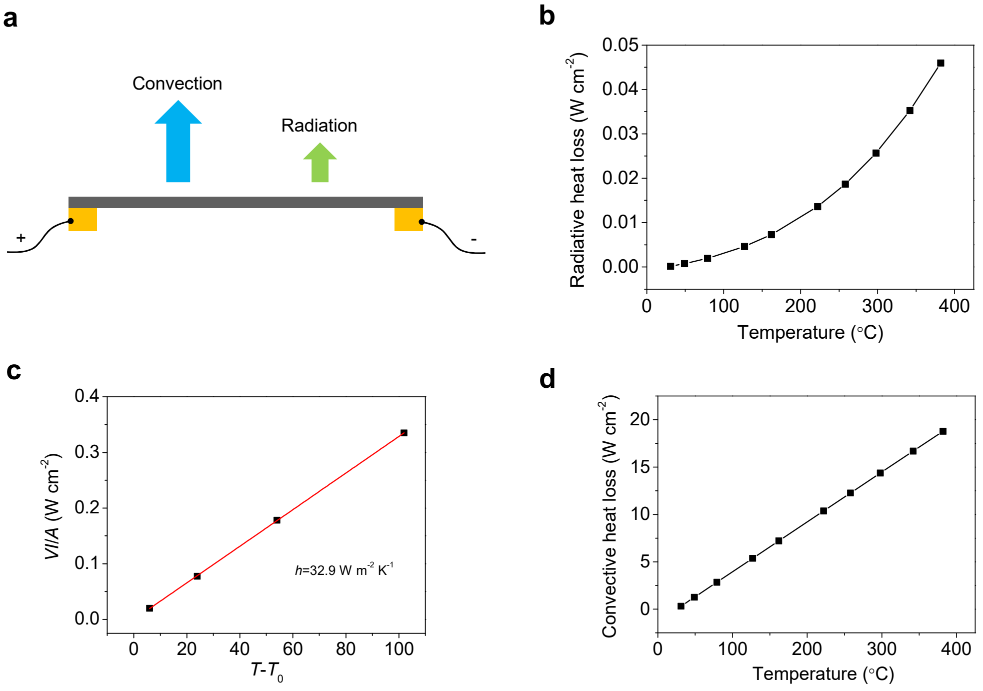

Supplementary Figure 13 (**a**) Schematic of the heat exchange between a free-standing GFF electrothermal heater and the surroundings. (**b**) Calculated radiative heat loss of the GFF heater as a function of temperature. (**c**) Fitting of the convective coefficient. (**d**) Convective heat loss as a function of temperature.

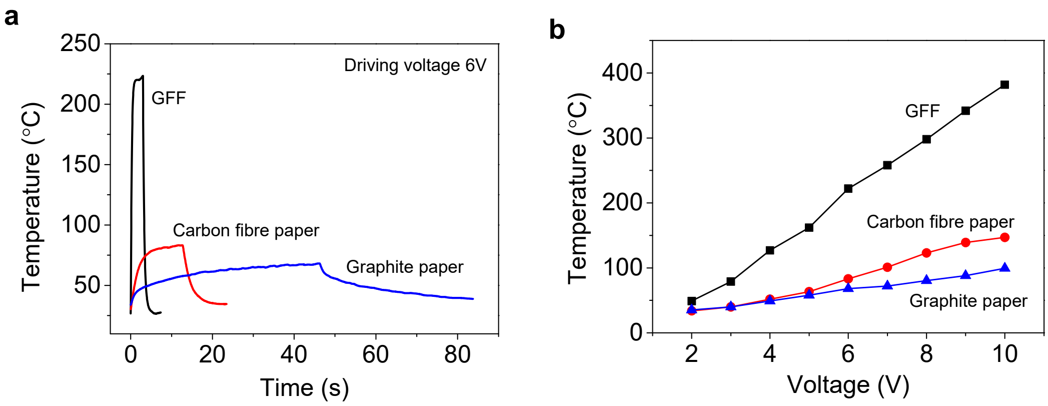

Supplementary Figure 14 Comparison of the electrothermal performance of GFF with commercial carbon fibre paper and graphite paper. (**a**) Temperature profiles of GFF, carbon fibre paper and graphite paper at 6 V. (**b**) Saturated temperature as a function of driving voltage. All the results were acquired in our lab while the three samples were of the same size (4×2 cm^2).

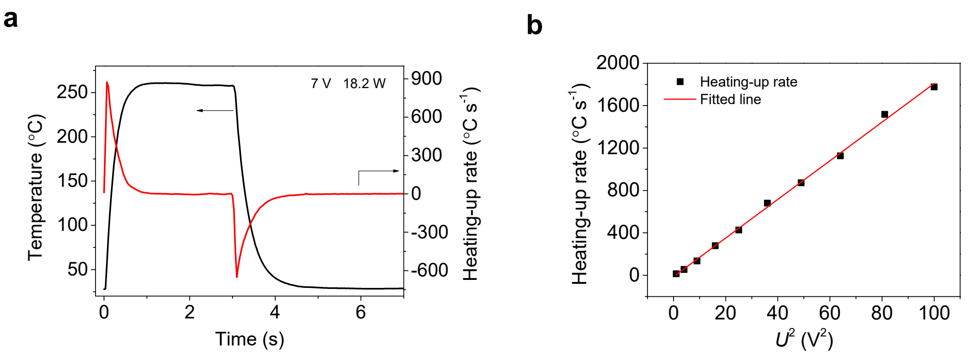

Supplementary Figure 15 (**a**) Temperature profile and change rate of temperature over time at an applied voltage of 7 V, the latter was derived by differentiating the temperature profile. (**b**) Plots and fitted line of maximum heating-up rate versus the square of applied voltage (U^2), indicating the maximum heating-up rate is linearly proportional to U^2.

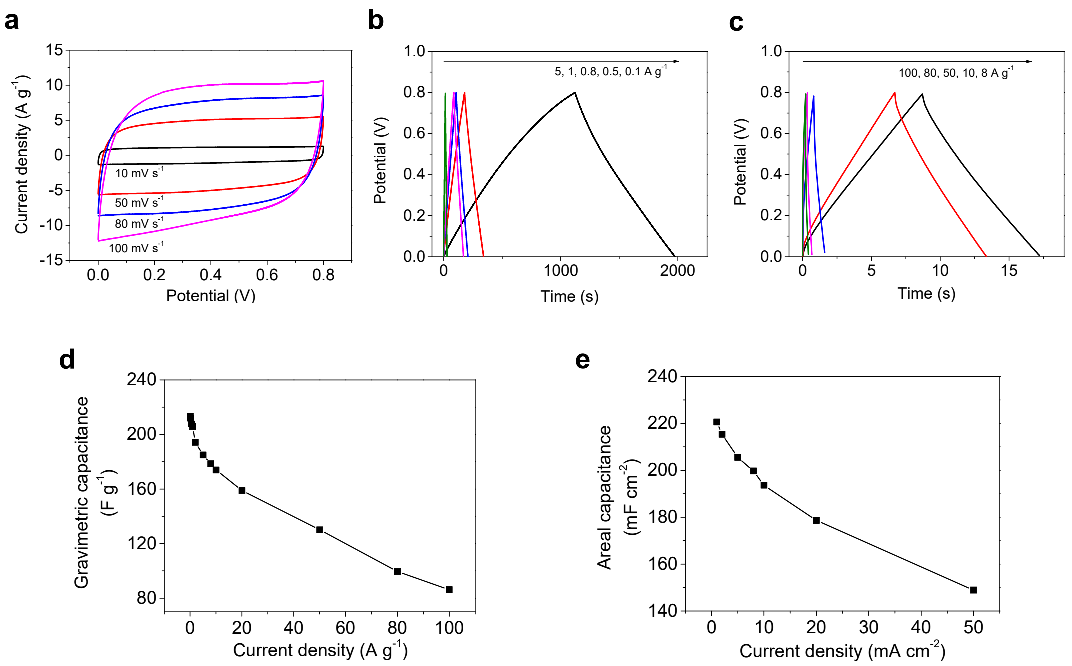

Supplementary Figure 16 Performance of the supercapacitors using 130GFF-N_2H_4 as electrodes. (**a**) CV curves of the 130GFF-N_2H_4 electrodes collected at scan rates of 10, 50, 80 and 100 mV s^{-1} in 1 M H_2SO_4 aqueous solution. Galvanostatic curves at various charging/discharging current densities (**b**) from 0.1 to 5 A g^{-1} and (**c**) from 8 to 100 A g^{-1}. (**d**) Gravimetric and (**e**) areal capacitance at different current densities.

Supplementary Table 1 Elemental analysis of GOFF, GFF-N_2H_4, and annealed GFFs.

	GOFF	GFF-N_2H_4	GFF-1000	GFF-2000	GFF-3000
C	66.56%	80.22%	95.48%	96.53%	98.04%
O	30.64%	10.81%	3.77%	2.37%	1.67%

Supplementary Table 2 Comparison of electrical and thermal conductivities with reported results.

Sample type	Density g cm^{-3}	Electrical conductivity S m^{-1}	Thermal conductivity W m^{-1} K^{-1}	Specific electrical conductivity	Specific thermal conductivity	Ref
130GFF-3000	0.22	2.8×10^4	3.0×10^2	1.3×10^5	1.4×10^3	This work
200GFF-3000	0.23	1.3×10^4	1.7×10^2	5.7×10^4	7.4×10^2	This work
MWNT buckypaper	Aligned 0.62	2×10^4	153	3.2×10^4	2.5×10^2	Ref 1
	Random 0.54	1.5×10^4	81	2.8×10^4	1.5×10^2	
High density CNT paper	0.77	3.2×10^4	-	4.2×10^4	-	Ref 2
	0.81	-	472	-	5.8×10^2	
	1.39	6.4×10^4	766	4.6×10^4	5.5×10^2	
rGO film	2.0	8.0×10^4	524	4.0×10^4	2.6×10^2	Ref 3
Functionalized graphene film	1.6	3.9×10^4	112	2.4×10^4	70	Ref 4
Annealed graphene film	2.03 (1800 °C annealed)	8.5×10^4	809.5	4.2×10^4	4×10^2	Ref 5
	2.1 (2200 °C annealed)	1.6×10^5	1238.3	7.6×10^4	5.9×10^2	
	2.0 (2850 °C annealed)	1.8×10^5	1434	9.0×10^4	7.2×10^2	
2000 °C annealed graphene film	-	1.0×10^5	1100	-	-	Ref 6
1000 °C annealed graphene film	-	1.3×10^3	61.8	-	-	Ref 7
Graphite nanoplatelet paper	1.15	-	178	-	1.5×10^2	Ref 8
2850 °C annealed graphene fibre	1.86	2.2×10^5	1290	1.2×10^5	6.9×10^2	Ref 9
Carbon nanofibre webs	-	490	-	-	-	Ref 10
Activated carbon fibre cloth/CNT	-	3200	-	-	-	Ref 11
Commercial carbon paper (Toray)	0.44 (TGP-H-060)	1.7×10^4	21	3.9×10^4	48	
	0.44 (TGP-H-090)	1.8×10^4	21	4.1×10^4	48	
	0.45 (TGP-H-120)	2.1×10^4	21	4.7×10^4	47	
Copper	8.5-8.9	6.0×10^7	401	$(6.7\text{-}7.1)\times10^6$	45-47	
Aluminum	2.7	4.0×10^7	237	1.5×10^7	88	

Supplementary Table 3 Average diameter of fibres in GOFFs and GFFs.

	130GOFF	130GFF-3000	200GOFF	200GFF-3000
Diameter (μm)	28.6±10.1	15.3±4.6	39.5±10.6	33.2±9.4

Supplementary Table 4 Comparison of the performance of 130GFFs (fibre length 2-5 mm) and SF130GFFs (fibre length 1-2 mm).

	Tensile strength (MPa)	Modulus (MPa)	Electrical conductivity ($S\ m^{-1}$)	Thermal conductivity ($W\ m^{-1}\ K^{-1}$)
130GOFF	0.7±0.2	37.6±3.8	$(2.0\pm0.3)\times10^{-3}$	-
SF130GOFF	0.6±0.1	35.7±4.1	$(1.9\pm0.4)\times10^{-3}$	-
130GFF-3000	0.6±0.1	29.9±6.4	$(2.83\pm0.14)\times10^{4}$	301.5±12.4
SF130GFF-3000	0.6±0.2	30.2±5.3	$(1.56\pm0.29)\times10^{4}$	210.3±11.3

Supplementary Table 5 Comparison of the Joule heating performance of GFFs with previously reported film heaters and commercial heating elements.

	Driving voltage (V)	Saturated temperature (°C)	Response time (s)	Ref
GFF	10	382	0.7	This work
Carbon fibre paper	10	147	8	This work
Graphite paper	10	99	42	This work
RGO film/PI	20	90	120	Ref 12
Graphene/glass	24	80	~300	Ref 13
$AuCl_3$-doped graphene/glass	10	80	~300	Ref 13
RGO/quartz	60	206	~120	Ref 14
RGO/PI	60	72	~10	Ref 14
Graphene-$AuCl_3$/PET	12	100	~100	Ref 15
Graphene-HNO_3/PET	12	65	~100	Ref 15
ITO	12	31.4	~100	Ref 15
SWNT/PET	12	80	60	Ref 16
Ag NW/PDMS	5	80	~60	Ref 17
Ag mesh/glass	9	128	~300	Ref 18
Ag NW-cloth	0.9	38	~300	Ref 19
CNT cloth	12	38	~300	Ref 19
PTC (positive temperature coefficient) heating plate	12	60-220		Commercial
MCH (metal ceramic heater) heating plate	10	130-340	>10	Commercial

The response time is denoted as the time required to reach the saturated temperature.

Supplementary Note 1 Analysis of the heat balance in GFF heaters.

The temperature of a heating system is a result of the balance between Joule heating and heat loss, while the former equals to the input power. Generally, the working process of a heater could be divided into three stages: heating-up, steady-state and cooling-down. The heat balance in each stage is expressed as:

$$cm \cdot \frac{dT}{dt} = VI - Q_c - Q_r \quad \text{(Heating-up)} \qquad \text{Supplementary Equation 1}$$

$$0 = VI - Q_c - Q_r \quad \text{(Steady-state)} \qquad \text{Supplementary Equation 2}$$

$$cm \cdot \frac{dT}{dt} = -Q_c - Q_r \quad \text{(Cooling-down)} \qquad \text{Supplementary Equation 3}$$

where c, m, T, t, V, I, Q_c and Q_r are specific heat capacity, mass, temperature of the sample, time, input voltage, current, convective heat loss and radiative heat loss, respectively. The convective heat loss is expressed by

$$Q_c = hA(T - T_0) \qquad \text{Supplementary Equation 4}$$

where h, A and T_0 are the convective coefficient, surface area of the sample and room temperature (25 °C), respectively. The radiative heat loss can be written as

$$Q_r = \varepsilon\sigma A(T^4 - T_0^4) \qquad \text{Supplementary Equation 5}$$

where ε is the surface emissivity of graphene (0.023)[13] and σ is the Stefan-Boltzmann constant (5.67×10^{-8} W m^{-2} K^{-1}).

In a simplified model for understanding the mechanism of heat exchange, we assumed A is approximately the sum of the upper and lower surface areas of the samples. Then the radiative heat loss could be calculated and is plotted in Supplementary Figure 13b. In the low temperature region (below 150 °C), the radiative heat loss (4.6×10^{-3} W cm^{-2} at 127 °C) is more than two orders of magnitude less than the input power (0.67 W cm^{-2} at 127 °C). Therefore the radiative heat loss is negligible while the temperature is below 150 °C and Supplementary Equation 2 can be written as:

$$VI = hA(T - T_0) \qquad \text{Supplementary Equation 6}$$

by linear fitting the equation, we obtained the convective coefficient h=32.9 W m^{-2} K^{-1} as the slope in Supplementary Figure 13c. Furthermore, the convective heat loss was thus obtained (Supplementary Figure 13d). The significantly higher values indicate that convection is the major heat loss that influences the temperature of GFF samples.

Supplementary References

1 Ding, W., Pengcheng, S., Changhong, L., Wei, W. & Shoushan, F. Highly oriented carbon nanotube papers made of aligned carbon nanotubes. *Nanotechnology* **19**, 075609 (2008).

2 Zhang, L., Zhang, G., Liu, C. & Fan, S. High-density carbon nanotube buckypapers with superior transport and mechanical properties. *Nano Lett.* **12**, 4848-4852 (2012).

3 Zhang, M. *et al.* Multifunctional pristine chemically modified graphene films as strong as stainless steel. *Adv. Mater.* **27**, 6708-6713 (2015).

4 Liang, Q., Yao, X., Wang, W., Liu, Y. & Wong, C. P. A three-dimensional vertically aligned functionalized multilayer graphene architecture: an approach for graphene-based thermal interfacial materials. *ACS Nano* **5**, 2392-2401 (2011).

5 Xin, G. *et al.* Large-area freestanding graphene paper for superior thermal management. *Adv. Mater.* **26**, 4521-4526 (2014).

6 Shen, B., Zhai, W. & Zheng, W. Ultrathin flexible graphene film: an excellent thermal conducting material with efficient EMI shielding. *Adv. Funct. Mater.* **24**, 4542-4548 (2014).

7 Renteria, J. D. *et al.* Strongly anisotropic thermal conductivity of free-standing reduced graphene oxide films annealed at high temperature. *Adv. Funct. Mater.* **25**, 4664-4672 (2015).

8 Xiang, J. & Drzal, L. T. Thermal conductivity of exfoliated graphite nanoplatelet paper. *Carbon* **49**, 773-778 (2011).

9 Xin, G. *et al.* Highly thermally conductive and mechanically strong graphene fibers. *Science* **349**, 1083-1087 (2015).

10 Qie, L. *et al.* Nitrogen-doped porous carbon nanofiber webs as anodes for lithium ion batteries with a superhigh capacity and rate capability. *Adv. Mater.* **24**, 2047-2050 (2012).

11 Dong, L. *et al.* Simultaneous production of high-performance flexible textile electrodes and fiber electrodes for wearable energy storage. *Adv. Mater.* **28**, 1675-1681 (2015).

12 Liu, Z. *et al.* Wet-spun continuous graphene films. *Chem. Mater.* **26**, 6786-6795 (2014).

13 Bae, J. J. *et al.* Heat dissipation of transparent graphene defoggers. *Adv. Funct. Mater.* **22**, 4819-4826 (2012).

14 Sui, D. *et al.* Flexible and transparent electrothermal film heaters based on graphene

materials. *Small* **7**, 3186-3192 (2011).

15 Kang, J. *et al.* High-performance graphene-based transparent flexible heaters. *Nano Lett.* **11**, 5154-5158 (2011).

16 Y.-H., Y. *et al.* Transparent film heater using single-walled carbon nanotubes. *Adv. Mater.* **19**, 4284-4287 (2007).

17 Hong, S. *et al.* Highly stretchable and transparent metal nanowire heater for wearable electronics applications. *Adv. Mater.* **27**, 4744-4751 (2015).

18 Kiruthika, S., Gupta, R. & Kulkarni, G. U. Large area defrosting windows based on electrothermal heating of highly conducting and transmitting Ag wire mesh. *RSC Adv.* **4**, 49745-49751 (2014).

19 Hsu, P.-C. *et al.* Personal thermal management by metallic nanowire-coated textile. *Nano Lett.* **15**, 365-371 (2015).
